# A longitudinal SARS-CoV-2 biorepository for COVID-19 survivors with and without post-acute sequelae

**DOI:** 10.1186/s12879-021-06359-2

**Published:** 2021-07-13

**Authors:** Stephanie M. LaVergne, Sophia Stromberg, Bridget A. Baxter, Tracy L. Webb, Taru S. Dutt, Kailey Berry, Madison Tipton, Jared Haberman, Benjamin R. Massey, Kim McFann, Omar Alnachoukati, Linda Zier, Thomas Heacock, Gregory D. Ebel, Marcela Henao-Tamayo, Julie Dunn, Elizabeth P. Ryan

**Affiliations:** 1 Department of Environmental and Radiological Health Sciences, Colorado State University, 1601 Campus Delivery, Fort Collins, CO 80523 USA; 2 Department of Food Science and Human Nutrition, Colorado State University, Fort Collins, CO USA; 3 Department of Clinical Sciences, Colorado State University, Fort Collins, CO USA; 4 Department of Microbiology, Immunology and Pathology, Colorado State University, Fort Collins, CO USA; 5 Department of Biomedical Sciences, Colorado State University, Fort Collins, CO USA; 6 University of Colorado Anschutz School of Medicine, Aurora, CO USA; 7 University of Colorado Health, Medical Center of the Rockies, Loveland, CO USA

**Keywords:** SARS-CoV-2, COVID-19, Post-acute sequelae of COVID-19 (PASC), Long-hauler, Biobank, Biorepository, Coronavirus

## Abstract

**Background:**

SARS-CoV-2 has swept across the globe, causing millions of deaths worldwide. Though most survive, many experience symptoms of COVID-19 for months after acute infection. Successful prevention and treatment of acute COVID-19 infection and its associated sequelae is dependent on in-depth knowledge of viral pathology across the spectrum of patient phenotypes and physiologic responses. Longitudinal biobanking provides a valuable resource of clinically integrated, easily accessed, and quality-controlled samples for researchers to study differential multi-organ system responses to SARS-CoV-2 infection, post-acute sequelae of COVID-19 (PASC), and vaccination.

**Methods:**

Adults with a history of a positive SARS-CoV-2 nasopharyngeal PCR are actively recruited from the community or hospital settings to enroll in the Northern Colorado SARS-CoV-2 Biorepository (NoCo-COBIO). Blood, saliva, stool, nasopharyngeal specimens, and extensive clinical and demographic data are collected at 4 time points over 6 months. Patients are assessed for PASC during longitudinal follow-up by physician led symptom questionnaires and physical exams. This clinical trial registration is NCT04603677.

**Results:**

We have enrolled and collected samples from 119 adults since July 2020, with 66% follow-up rate. Forty-nine percent of participants assessed with a symptom surveillance questionnaire (*N* = 37 of 75) had PASC at any time during follow-up (up to 8 months post infection). Ninety-three percent of hospitalized participants developed PASC, while 23% of those not requiring hospitalization developed PASC. At 90–174 days post SARS-CoV-2 diagnosis, 67% of all participants had persistent symptoms (*N* = 37 of 55), and 85% percent of participants who required hospitalization during initial infection (*N* = 20) still had symptoms. The most common symptoms reported after 15 days of infection were fatigue, loss of smell, loss of taste, exercise intolerance, and cognitive dysfunction.

**Conclusions:**

Patients who were hospitalized for COVID-19 were significantly more likely to have PASC than those not requiring hospitalization, however 23% of patients who were not hospitalized also developed PASC. This patient-matched, multi-matrix, longitudinal biorepository from COVID-19 survivors with and without PASC will allow for current and future research to better understand the pathophysiology of disease and to identify targeted interventions to reduce risk for PASC. Registered 27 October 2020 - Retrospectively registered, https://clinicaltrials.gov/ct2/show/NCT04603677.

## Background

Since its initial identification in December 2019, severe acute respiratory syndrome-coronavirus-2 (SARS-CoV-2) has caused a global pandemic, killing millions and infecting over 100 million individuals worldwide [1, 2]. SARS-CoV-2 leaves behind millions of survivors, most of whom recover, but for many, symptoms persist [3–7]. A broad range of symptoms can last over 6 months, and can include persistence of initial acute symptoms or development of new symptoms [3, 8, 9]. Persistent or new symptoms affecting COVID-19 survivors are named post-acute sequelae of COVID-19 (PASC) [9, 10]. Risk factors for developing PASC are currently being defined globally. A systematic and calculated approach to investigating the course of SARS-CoV-2 infection is needed to understand varied susceptibility to severe SARS-CoV-2 disease and COVID-19 sequelae. Scientists and industry have expressed the importance of a biorepository as an essential building block for critical research [11–17]. Biobanking efforts for SARS-CoV-2 are underway globally, yet most are comprised of limited specimen types collected from participants at a single time point [16, 18–21]. The Northern Colorado SARS-CoV-2 Biorepository (NoCo-COBIO) is uniquely multifaceted and contains longitudinal clinical data, electronic medical records (EMR), demographics, quality of life surveys, and biological specimens of stool, saliva, nasopharyngeal swab specimens, plasma, serum, peripheral blood mononuclear cells (PBMCs), and breastmilk if available, from each SARS-CoV-2-infected participant. The biospecimens are immensely valuable to longitudinally evaluate mechanisms underlying persistent symptoms and PASC. This clinically integrated-biorepository and COVID-19 survivorship project has been successful in securing patient compliance to long-term follow-up visits with the study team, and is a powerful resource for global collaboration and co-creation of statistically-powered study designs for assessing molecular, immune, and metabolic signatures associated with PASC in adults [22].

## Methods

### Participant identification and enrollment

The identification and enrollment of participants into NoCo-COBIO is a joint effort between Colorado State University (CSU), a land grant institution with a coordinated state-wide extension system, and the University of Colorado Health System (UCH). Individuals are eligible for participation if they have had a positive SARS-CoV-2 polymerase chain reaction (PCR) test and are at least 18 years of age. Participants are recruited from the community via the health department screening, local medical clinics, emails, recruitment flyers, web-based announcements, and directly through UCH northern Colorado hospitals, including Poudre Valley Hospital (PVH) in Fort Collins, Medical Center of the Rockies (MCR) in Loveland, and Greeley Hospital in Greeley. The UCH Trauma Research Department (TRD) identifies eligible hospitalized patients through Epic, the EMR platform. UCH clinicians that oversee patients at the northern Colorado hospitals are aware of the biorepository and may refer potential participants to the biobank research team for follow-up. UCH staff directly approach eligible, hospitalized patients on the ward for consent and enrollment. The UCH investigators also conduct frequent EMR searches to recruit recently discharged/diagnosed COVID-19 patients. Additionally, enrolled participants have also assisted via word-of-mouth and personal networks.

This biorepository was approved by CSU’s Research Integrity and Compliance Review Office Institutional Review Board (IRB; protocol ID 20-10063H), as well as UCH IRB (Colorado Multiple IRB 20–6043) and is registered with ClinicalTrials.gov (NCT04603677). All enrolled participants provide written informed consent. Adults with no history of infection and with a negative SARS-CoV-2 PCR test are enrolled for the same study visit and specimen collections. Given that these individuals are not matched to infected individuals, they were not included in the comparative analysis. All participants receive $25 cash compensation at each of the four study visits.

### Clinical data

All data is de-identified and password-protected for analysis. Clinical data obtained from the EMR are stored in Research Electronic Data Capture (REDCap), and each record is assigned a unique identifier. Demographic data that may affect COVID-19 outcomes [23–26], such as socioeconomic status, employment status, ethnicity, race, and clinical data are obtained from participants at their clinic visits. A physician performs a physical exam at each outpatient visit. Participants are categorized as having a mild, moderate, or severe initial infection based on oxygen requirements during their acute illness (no oxygen requirement, 1-5 L oxygen requirement, and greater than 5 L oxygen use, respectively). During follow-up visits, participants assessed with a survey of 70 symptoms to identify new or persistent sequelae of COVID-19 (Table 1**).** Patients are defined as having PASC if they experience at least one of the following symptoms commonly associated with PASC: fatigue, dyspnea, joint pain, chest pain, or cognitive impairment, at any follow-up visit [27]. Fatigue was not described nor defined for the participants during administration of the post-acute symptom/sequelae questionnaire and was subject to each participant’s interpretation. However, if the participant described excessive tiredness or difficulties with physical activity that was worse than their baseline state of health prior to COVID-19 infection, then the participant was marked as having fatigue. Dyspnea was defined for participants as difficulty breathing or shortness of breath.

**Table 1:** Symptom surveillance across organ systems for study participants during longitudinal evaluations and in accordance with biospecimen collections

System	Symptom
General	Fever, fatigue, exercise intolerance, weakness, appetite loss, hair loss, night sweats
Neurologic	Headache, dizziness, loss of consciousness, deafness or change in hearing, numbness, tingling, or burning sensation in extremities, loss of taste, loss of smell, change in taste, forgetful or absent minded, confused, difficulty concentrating
Ocular	Eye pain, redness, tearing, light sensitivity, floaters, loss of vision, blurry vision, flashes of light, eye burning
Head and Neck	Sore throat, runny nose, congestion, sinusitis, facial pressure, neck pain, mouth sores
Cardiac	Palpitations, chest pain, lower extremity swelling, chest burning, chest pressure, rapid heart rate
Pulmonary	Difficulty breathing, cough, phlegm in the back of throat, wheezing
Gastrointestinal	Abdominal pain, diarrhea, constipation, nausea, vomiting, reflux
Integumentary	Rash, dry skin, itching, jaundice
Musculoskeletal	Muscle aches, body aches, back pain, joint pain
Reproductive	Erectile dysfunction, amenorrhea, change in menstruation
Hematologic	Easy bruising, bleeding, clot
Psychologic	Anxiety, depression, difficulty sleeping
Other	Patients are asked about other persistent or new symptoms not mentioned above

The study team administers the Rand SF-36 item health survey (SF-36) at two clinical follow-up visits to assess social impacts and quality of life after COVID-19. This survey gives an average maximum score of 100 in 8 different categories: general health, physical functioning, role limitations due to physical health, role limitations due to emotional health, energy level, emotional wellbeing, social functioning, and pain. A total, combined score is also given, with a maximum of 3600. The survey asks questions about the participants’ emotional and physical well-being during the 4 weeks prior to survey administration. This validated instrument [28, 29] also compares current health to their health 1 year prior, as well as reports on the expectations for future health outcomes.

### Biospecimen collection and transport

Participants consent to provide all biospecimens at 4 visits over an enrollment period of 6 months. The approximate time points for each of the study visits with symptom and specimen collections are: at the time of enrollment (Baseline Visit 1), 1 month after baseline (Visit 2), 3 months after baseline (Visit 3), and 6 months after enrollment (Visit 4). The second and third visits may take place in the hospital if the participant remains hospitalized. Follow-up visits take place at designated campus clinical research labs, medical clinic sites or in some specialized cases at home. After 6 months, participants may provide additional consent for a one-year follow-up visit.

**Fig. 1:**
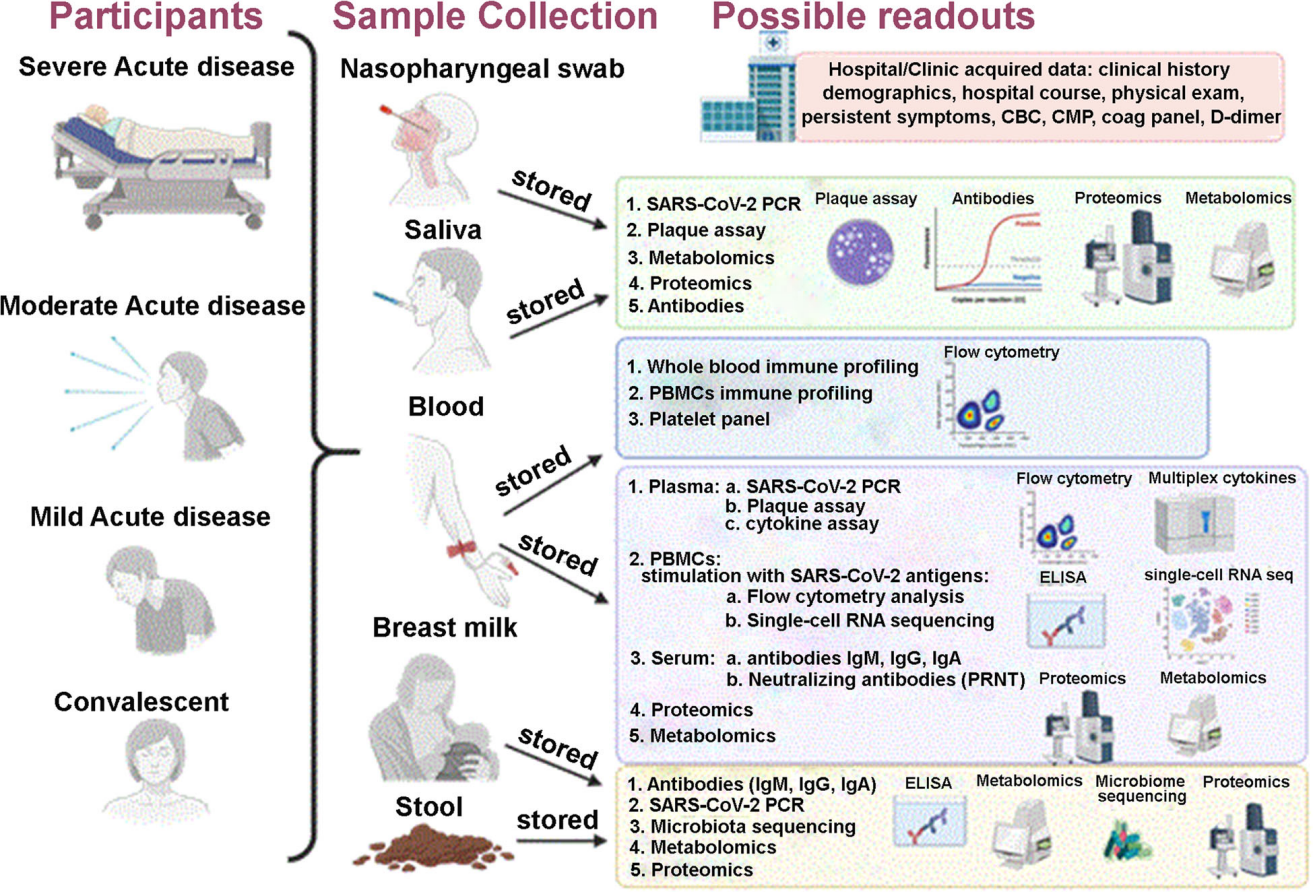
Schematic of multi-matrix biorepository, sample processing, analysis and storage from adults across the clinical spectrum of disease severity. Image was created using BioRender.com (Toronto, ON, Canada). PBMCs denotes peripheral blood mononuclear cells, CBC complete blood count, CMP comprehensive metabolic panel, Coag coagulation

Figure 1 depicts the trajectory of each sample collected. Participants provide approximately 50 mL of blood, 10 mL of saliva (5 mL in viral transport media (VTM, sterile hanks balanced salt solution (ThermoFischer Scientific) with 2% FBS) for viral propagation and 5 mL without VTM), a stool sample, and a nasopharyngeal swab specimen at each visit. Blood is collected into five 8 mL sodium citrate cell preparation tubes (CPTs) (BD BioSciences, Franklin Lakes, NJ) and one 5 mL serum separator tube (VWR). Saliva samples are collected by expectoration, or a tracheal aspirate specimen is collected if participants are intubated. Samples with VTM are kept on ice during transport. Nasopharyngeal swab specimens are collected with a latticed nasal swab (Resolution Medical). The swab is then placed in VTM and kept on ice for transport. Stool is either self-collected by the participants or collected by the participant’s nurse if they are hospitalized. The stool can be collected prior to the study visit and stored frozen. Breast milk is collected from lactating participants, either fresh in pre-distributed breast milk storage bags, or previously pumped and stored frozen.

Each patient sample is de-identified and labeled with a unique study code identifier. Information linking participant identification to the study codes is stored in the offices of the investigators and will be maintained for up to 5 years following the cessation of the study. Nasopharyngeal swab and saliva in VTM from acutely infected patients are transported to and processed in CSU’s biosafety level-3 (BSL-3) facilities. Blood, nasopharyngeal samples, and saliva collected from convalescent patients and all fecal samples are transported to and processed in biosafety level-2 (BSL-2) facilities in accordance with CSU’s IRB and Centers for Disease Control and Prevention (CDC) recommendations. Transportation of specimens from clinical sites to laboratory facilities is done in accordance with U.S. Department of Transportation hazardous materials regulations.

### Whole blood processing protocol

An aliquot of whole blood is removed from a CPT, incubated with platelet surface markers, and analyzed by flow cytometry with LSR II (BD Biosciences) and FACSAria Cell Sorter (BD Biosciences) instruments. A second aliquot of blood is removed from the CPT and stained for leukocytes and endothelial cells and analyzed by flow cytometry on a Cytek Aurora (Cytek Biosciences) instrument. The CSU Flow Cytometry Core has instruments with the same laser configurations inside and outside the BSL-3 facility to preserve consistent results when analyzing samples in different locations.

The CPTs are then centrifuged according to manufacturer instructions. The plasma is removed, and the PBMCs are separated and washed. A small aliquot of PBMCs is removed for additional antibody staining and flow cytometry analysis. PBMCs are cryostored in freezing media (90% heat inactivated fetal bovine serum (FBS) (Avantor) and 10% dimethyl sulfoxide (DSMO) (Sigma Altrich)). PBMC are first stored in an isopropanol-filled Nalgene® Mr. Frosty (Thermo Scientific) at − 80 °C for 24 h and transferred into liquid nitrogen for long-term cryopreservation.

### Plasma and serum aliquots

Following removal from CPTs, plasma is stored in 1 mL aliquots at − 80 °C. Blood collected in serum separator tubes is allowed to clot for 30 min, and then spun at 1000 x g at 4 °C for 10 min. Serum is removed and stored in 300 uL aliquots at − 80 °C. Each participant’s samples are designated to an individual cryobox and organized by their visit for quick sample retrieval.

### Saliva and nasopharyngeal processing

An aliquot of both saliva and nasopharyngeal sample in VTM are sent for quantitative PCR (qPCR) analysis for SARS-CoV-2. The remaining saliva is centrifuged, the pellet is discarded, and the supernatant is aliquoted for − 80 °C storage. Nasopharyngeal samples are aliquoted and stored at − 80 °C for SARS-CoV-2 viral titer analysis using plaque assay.

### Stool processing

Stool samples are aliquoted and stored at − 80 °C for DNA, RNA, secretory immunoglobulin A, and metabolite analysis.

### Statistical analysis

Statistical analysis was performed using Fischer’s Exact Test.

## Results

To date, 119 participants have been enrolled (Table 2). Thirty-one participants are classified as having had a severe initial infection (defined as oxygen requirement greater than 5 L/min), 30 experienced a moderate infection (defined as oxygen requirement of 1-5 L/min), and 58 experienced a mild or asymptomatic disease course (no oxygen required).

**Table 2:** Characteristics of the study participants at baseline (*N* = 119)

Characteristics	Mild (***n*** = 58)	Moderate (***n*** = 30)	Severe (***n*** = 31)
Age, mean (SD), y	39.2 (15.9)	62.1 (14)	59.7 (13.3)
Sex, no. (%)			
Female	42 (71)	15 (52)	9 (29)
Male	16 (29)	15 (48)	22 (71)
BMI^a^, mean (SD)	26.3 (5.3)	36.5 (7.5)	36.5 (10.7)
Ethnicity, no. (%)			
Non-Hispanic/Latinx	54 (93)	23 (76.6)	18 (58)
Hispanic/Latinx	4 (7)	7 (23.4)	13 (42)
Co-existing conditions, no. (%)			
CVA	0	3 (11)	1 (3.2)
COPD	1 (2)	5 (18.5)	3 (9.7)
DM	1 (2)	8 (26)	16 (48.4)
HTN	1 (2)	11 (40)	19 (57.6)
CAD	1 (2)	2 (7.4)	1 (3.2)
Active cancer	0	3 (11)	0

^a^*BMI* denotes body mass index, *CVA* cerebrovascular accident, *COPD* chronic obstructive pulmonary disease, *DM* diabetes mellites, *HTN* hypertension, *CAD* coronary artery disease

Sixty-six percent (*N* = 79) of all participants have completed 2 visits, 50% (*N* = 59) have completed 3 visits, and 33% (*N* = 39) have completed 4 visits. Including follow-ups, 301 visits have been conducted, and at 88% of these visits, participants were able to provide all samples requested. Participant visits are conducted at the time of enrollment (Visit 1-Baseline), and at 1 month (Visit 2), 3 months (Visit 3), and 6 months (Visit 4) from the time of enrollment. Nearly every visit or study encounter has yielded blood, nasopharyngeal, and saliva samples (99, 99, and 98% respectively), and 88% of visits yielded stool samples. The team typically processes 10 sample sets in a single day. The time to obtain, process, and store samples from 10 participants is approximately 50 personnel hours (5–7 people) and costs $1500 U.S. dollars per sample.

The participants included in the biorepository thus far represent a broad age range (18–82 years), and the elderly were more likely to have moderate or severe COVID-19 (Table 2). Seventy-one percent of participants are females, and 29% are males. Of participants that suffered severe disease, 69% are males. Nineteen participants identify as Hispanic or Latino, and of these, 15 were hospitalized with COVID-19. Obese participants in our cohort were also more likely to suffer from moderate or severe COVID-19. The average body mass index (BMI) of participants with mild disease was 26, in contrast to an average BMI of 36.5 in both the moderate and severe groups (Table 2).

**Fig. 2:**
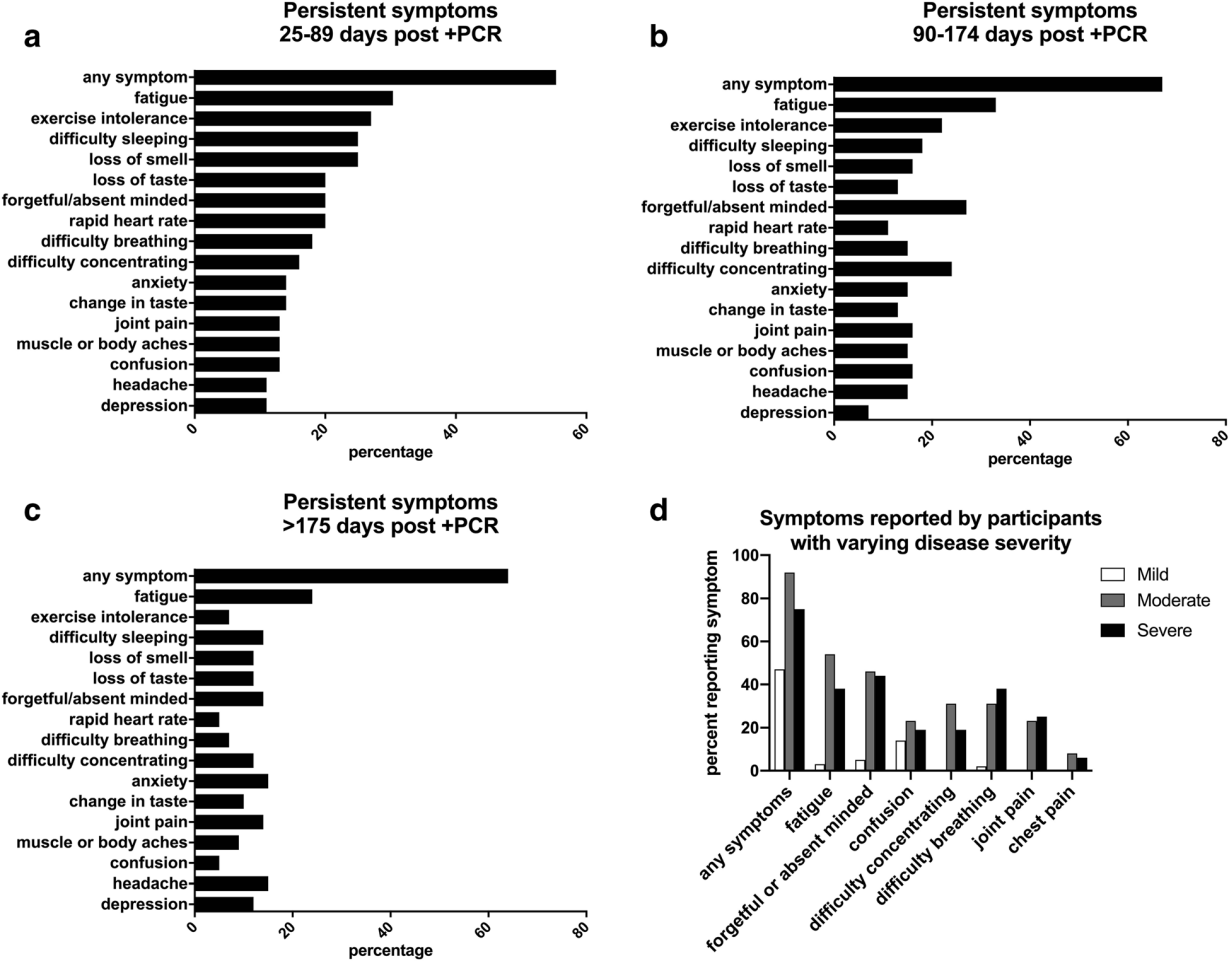
Percentage of participants reporting symptoms between 25 and > 175 days post infection. **a** 25–89 days (*N* = 56), **b** 90–174 days (*N* = 55), and **c** greater than 175 days (*N* = 42). The number of participants in the surveys vary for each time point. **d** Percentage of participants who suffered mild (*N* = 43), moderate (*N* = 13), or severe (*N* = 16) COVID-19, reporting symptoms at any follow-up visit (from days 20–243 post infection)

PASC was defined herein as having at least one common persistent symptom, and has been previously described in the literature [3, 6, 27] for including fatigue or tiredness, dyspnea, difficulty breathing, or shortness of breath, chest pain, joint pain, and perceived cognitive impairment such as “brain fog”, absent-minded, forgetfulness, difficulty concentrating or confusion) at any follow-up visit. Of the 79 participants who have completed follow-up visits, 75 have completed the persistent symptoms questionnaire. Forty-nine percent of participants who followed up (*N* = 37 of 75) had PASC at any point during their follow-up visits, with symptom surveillance performed at up to 8 months from infection. Significantly more hospitalized participants (93%, *N* = 25 of 27) developed PASC compared to 23% of those not requiring hospitalization (*N* = 11 of 47) (*p* = 0.0001). Participants who had moderate and severe COVID-19 were equally as likely to develop PASC (92.3 and 92.9%, respectively). At 25–89 days post positive SARS-CoV-2 PCR, 55% of participants who attended clinic (*N* = 31 of 56) had at least one persistent symptom attributed to COVID-19 (Fig. 2a). At 90–174 days post positive SARS-CoV-2 PCR, 67% (*N* = 31 of 49) of participants attending clinic had persistent symptoms (Fig. 2b). Sixty-four percent of all participants (Fig. 2c), and 90% of hospitalized patients still reported symptoms after 175 post initial infection, with the most symptoms reported in this group being fatigue, difficulty concentrating, absent-minded or forgetful (also described as brain fog), ageusia, and anosmia. Participants with moderate disease reported more symptoms than participants with mild or severe disease, however those with severe disease were more likely to experience difficulty breathing and joint pain (Fig. 2d).

The individuals included in this biorepository also have a variety of underlying health conditions that have been associated with severe COVID-19, including, but not limited to, obesity, stroke, diabetes mellitus, liver disease, connective tissue disease, chronic obstructive pulmonary disease (COPD), congestive heart failure, and coronary artery disease [23, 24, 30–33]. In our cohort, PASC is more prevalent in those with obesity and advanced age. This may be confounded by disease severity, given most participants with severe COVID-19 are obese and elderly, and a larger sample size is needed to correct for bias. When comparing those with and without PASC in participants with mild disease only, there was no significant difference in age, gender, BMI, or underlying health conditions. Interestingly, nearly all patients with PASC had normal physical exams.

**Fig. 3:**
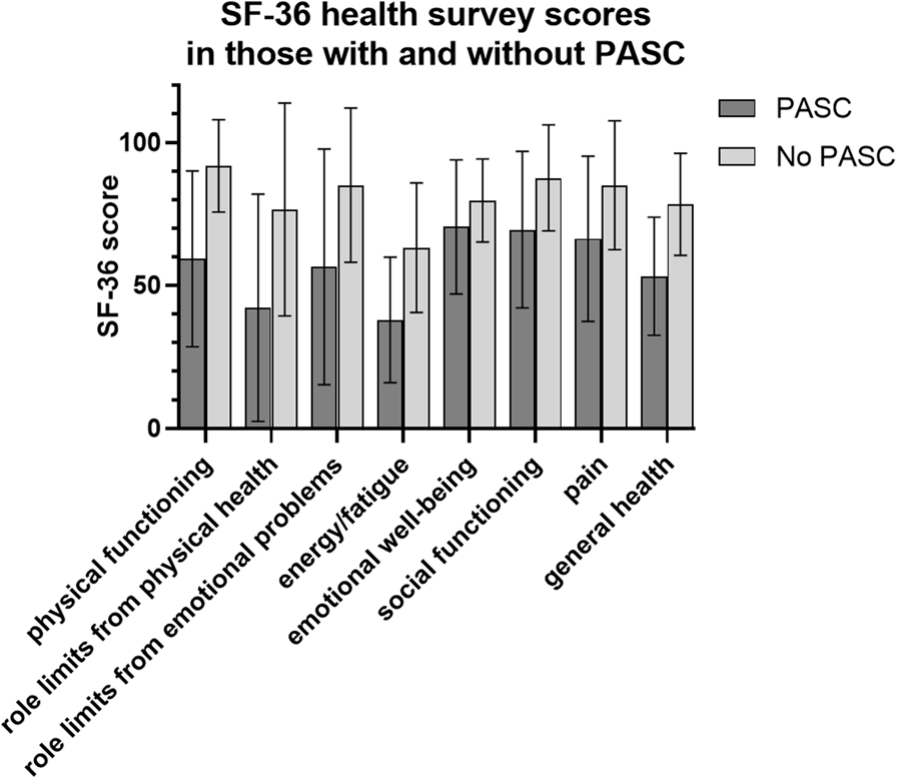
Rand SF-36 item health survey scores in participants with and without Post-Acute Sequelae of COVID-19 (*N* = 69). The survey was administered an average of 125 days post SARS-CoV-2 positive PCR diagnostic test result

SF-36 surveys were performed in 69 participants: 33 with PASC and 36 without PASC. The survey was performed at any point between day 21 and day 288 after positive SARS-CoV-2 PCR, with a mean of day 125. Participants with PASC had mean total score of 2019 out of a maximum score of 3600, while those without PASC had a higher mean total score of 2934. On average, participants with PASC scored lower in each of the 8 individual categories measured with SF-36 when compared to those without PASC. Questions in each of the 8 categories are averaged to give a maximum score of 100 for each category. The individual score differences between those with PASC and without are as follows: physical functioning (59 vs 92), role of limitations due to physical health (42 vs 77), role of limitations due to emotional problems (57 vs 85), energy and fatigue (38 vs 63), emotional well-being (71 vs 80), social functioning (70 vs 87), pain (66 vs 85), and general health (53 vs 79) (Fig. 3**)**.

## Discussion

Although challenging to implement and establish, longitudinal SARS-CoV-2 biobanking efforts are a valuable investment and resource to expedite research and further our knowledge of COVID-19 and PASC [11, 22, 34]. In this cohort, obesity, age, male sex, and Hispanic/Latinx ethnicity were risk factors for severe COVID-19. These clinical characteristics have been previously associated with severe disease [35–37], validating the NoCo-COBIO cohort. Hospitalized patients were also significantly more likely to suffer from PASC than non-hospitalized participants, similar to what has been previously reported for PASC [4, 5, 7]. The duration of PASC in this prospective cohort analysis is extensive, and we found that symptoms persisted in 89% of hospitalized participants at 90–174 days post infection, and in 90% after 174 days. Some of these participants required prolonged hospital stay, and many symptoms could be attributed to injuries or sequelae sustained from ICU stay alone [38]. However, those hospitalized with moderate disease (oxygen requirement of 1-5 L/min, not requiring ICU stay) were equally as likely to develop PASC as those who had severe disease (oxygen requirement of greater than 5 L/min). Twenty-three percent of our participants who were not hospitalized for COVID-19 also developed PASC. Although those who suffered mild COVID-19 are not at as high of a risk for developing PASC as those who were hospitalized, a staggering amount of people could be affected by PASC given high infection rates in the population. We have enrolled adults with no history of SARS-CoV-2 infection for matched biospecimens (*N* = 17). These non-infected individuals also completed the symptom questionnaire and did not report any symptoms. This is important, given some symptoms of PASC such as anxiety or fatigue could be perceived as attributable to social isolation and decreased activity during the pandemic. The adults with PASC had lower quality of life scores on SF-36 surveys when compared to those without PASC, consistent with previous research [39]. PASC could further burden our health care system during an already strained pandemic time, and continued prospective follow-up is essential to understand the pathophysiology underlying the physical difficulties and emotional complications of PASC [40, 41]. We aim to further analyze our cohort to find immunologic, metabolic, microbial, proteomic, and long-term clinical differences in those with and without PASC. Physical examinations and symptom surveillance over time increase the significance of this biorepository as it allows for depth during examination of those with post-viral sequelae. The administration of quality-of-life questionnaires is an additional unique aspect of this protocol, providing a structured tool to further assess those with PASC. This multifaceted approach to post-viral symptom surveillance is of important correlative and potential causal biological value with the linkages to innumerable data points and analytes that are under investigation from the specimens.

Though we were able to include individuals with Hispanic or Latinx ethnicity, the lack of racial diversity is one major shortcoming of the biorepository and we aim to enroll participants of various races to the biobank. Another shortcoming is the lack of a control group. The non-infected adults enrolled were healthier and younger than the COVID-19 infected participants. We do seek to enroll a participant-matched control group for age, BMI, sex and other co-morbidity characteristics. Additionally, the Cameron Peak wildfire was a major disturbance to air quality and outdoor activity in northern Colorado during the August–October 2020 portion of our data collection, which may have affected health perception and symptoms.

This biobank protocol with a manual of operations and standard operating procedures provides a template for other academic-clinical teams that reside outside of traditional metropolitan settings to replicate for ensuring compliance and follow-up for patient-matched, multi-matrix biospecimens. These stored sample sets are needed for improving diagnostic designs, advancing therapeutics research and development of SARS-CoV-2 vaccines, as well as understanding those with prolonged course of illness and those without PASC.

## Conclusions

People have diverse and complex courses of illness from COVID-19 across organ systems. In this cohort, patients who were hospitalized for COVID-19 were more likely to have PASC than those not requiring hospitalization, yet the 23% of patients who were not hospitalized and developed PASC also merit research attention and quality of life assessments. This patient-matched, multi-matrix, longitudinal biorepository from COVID-19 survivors will allow for current and future research to better understand the pathophysiology of disease, prolonged symptoms and to identify targeted interventions to reduce risk for PASC.

## Data Availability

All data available for this study are presented in the manuscript. Additional information regarding the status of the biorepository and continuous enrollment is available on the trial registration site.

## Abbreviations

PASC: Post-acute sequelae of COVID-19
NoCo-COBIO: Northern Colorado SARS-CoV-2 Biorepository
SARS-CoV-2: Severe acute respiratory syndrome-coronavirus-2
EMR: Electronic medical records
PBMCs: Peripheral blood mononuclear cells
CSU: Colorado State University
UCH: University of Colorado Health System
PCR: Polymerase chain reaction
PVH: Poudre Valley Hospital
MCR: Medical Center of the Rockies
TRD: Trauma Research Department
IRB: Institutional Review Board
REDCap: Research Electronic Data Capture
SF-36: Rand 36 health survey
VTM: Viral transport media
CPTs: Cell preparation tubes
BSL-3: Biosafety level-3
BSL-2: Biosafety level-2
CDC: Centers for Disease Control and Prevention
FBS: Fetal bovine serum
DSMO: Dimethyl sulfoxide
qPCR: Quantitative PCR
BMI: Body mass index
COPD: Chronic obstructive pulmonary disease
CBC: Complete blood count
CMP: Comprehensive metabolic panel
Coag: Coagulation
